# Effect of interventions for non-emergent medical transportation: a systematic review and meta-analysis

**DOI:** 10.1186/s12889-022-13149-1

**Published:** 2022-04-21

**Authors:** Paul G. Shekelle, Meron M. Begashaw, Isomi M. Miake-Lye, Marika Booth, Bethany Myers, Andrew Renda

**Affiliations:** 1 West Los Angeles Veterans Affairs Medical Center, 11301 Wilshire Blvd., Los Angeles, CA 90073-1003 USA; 2 RAND Corporation, Southern California Evidence-Based Practice Center, 1776 Main St, Santa Monica, CA 90401-3208 USA; 3 Louise M. Darling Biomedical Library, University of California Los Angeles (UCLA), 12-077 Center for Health Sciences, Los Angeles, CA 90095 USA; 4 Humana, Inc, 500 W. Main Street, HUM 14, Louisville, KY 40202 USA

**Keywords:** Non-emergency medical transportation, Health outcomes, Systematic review

## Abstract

**Introduction:**

Transportation is an important social determinant of health. We conducted a systematic review of the associations on health and health care utilization of interventions aimed at reducing barriers to non-emergency transportation and non-medical transportation.

**Methods:**

We searched three databases and the gray literature through mid-January 2022. Included studies needed to assess an intervention targeted at non-emergency or non-medical transportation barriers, report missed (or kept) visits, health care utilization, costs, or health outcomes. Data extraction was performed in duplicate and included information about study design, results, and risk of bias. Primary outcomes were frequency of missed appointments, health care utilization, costs, and health outcomes. Synthesis was both narrative and meta-analytic using a random effects model.

**Results:**

Twelve studies met inclusion criteria, three randomized trials, one controlled trial, and eight observational studies. All included studies had some element of risk of bias. Populations studied usually had chronic or serious health conditions or were poor. Interventions included van rides, bus or taxi vouchers, ride-sharing services, and others. Meta-analysis of seven studies (three trials, four observational studies) yielded a pooled estimate of missed appointments = 0.63 (95% confidence interval [CI] 0.48, 0.83) favoring interventions. Evidence on cost, utilization, and health outcomes were too sparse to support conclusions. Evidence on the effect of non-medical transportation is limited to a single study.

**Conclusions and relevance:**

Interventions aimed at non-emergency transportation barriers to access health care are associated with fewer missed appointments; the association with costs, utilization or health outcomes is insufficiently studied to reach conclusions.

This review was registered in PROSPERO as ID CRD42020201875.

**Supplementary Information:**

The online version contains supplementary material available at 10.1186/s12889-022-13149-1.

## Introduction

Social determinants of health are receiving increasing attention as a target for interventions to try and improve health outcomes. Transportation barriers are one category of social determinants. Transportation barriers can be categorized as Emergency Medical Transport (EMT), Non-Emergent Medical Transport (NEMT), and Non-Medical Transport (NMT). Transport for medical emergencies typically happens through the 911 system and occurs in ambulances with trained personnel. Non-emergency transport can be provided by people without medical training and occur in vans or cars. Barriers to NEMT and NMT can be assessed with validated screening instruments (such as the Accountable Health Communities Health-Related Social Needs Screening Tool question “In the past 12 months, has lack of reliable transportation kept you from medical appointments, meetings, work or from getting things needed for daily living?”) [1] NEMT barriers have been estimated to cause foregone or delayed care in up to 3.6 million people annually and be responsible for 25% or more of missed clinic appointments [2, 3]. More than 20% of older adults, and even more with chronic diseases, do not drive [4]. This means these persons also have barriers to non-medical transport such as shopping and social engagements, which may also be deleterious to health. Medicaid and Medicare Advantage plans show increasingly widespread use of non-emergency medical transportation (NEMT), via Medicaid home and community-based services (HCBS) and Medicare Advantage supplemental benefits [5]. Solutions include company-contracted transportation services, as well as vouchers for other public transportation options. The arrival of rideshare programs (such as Uber and Lyft) have been embraced as a new option to overcome transportation barriers [6]. The current COVID pandemic further exacerbates NEMT and NMT challenges, as public transportation options have stopped or operated at reduced schedules. A prior review, whose search was performed in January 2018, identified 10 studies meeting inclusion criteria, which were broad and included multicomponent interventions where the transportation component may have been small and/or unstated in size [7]. Since 2018 new studies have been published, which focused primarily on transportation barriers. In order to better understand NEMT and NMT interventions studies and their effects, we conducted a systematic review of published and gray literature studies of interventions for transportation, both non-emergency medical and non-medical, on utilization, costs, and health outcomes in children and adults.

## Methods

This topic was developed in consultation with the sponsor, Humana. This review was registered in PROSPERO as ID CRD42020201875. It is reported according to the PRISMA guidelines [8].

### Data sources and searches

The search strategy, including the search terms and databases used, was created by an experienced reference librarian. We conducted searches in PubMed and Cochrane Review and Trials and Web of Science core collection from inception to 01/14/2022. The searches used included “health services accessibility,” “appointments and schedules,” “transportation of patients,” and “rideshare” as the set of terms. See Additional file 1: Appendix A for complete search strategy. In addition, we searched the Social Interventions Research & Evaluation Network (SIREN) database on 01/26/21 using their Social Determinant of Health ‘Transportation’ filter categorization. SIREN is a University of California, San Francisco project focused on catalyzing high quality research, collecting, summarizing, and disseminating research, and increasing capacity to evaluate social determinants of health interventions [9]. We searched for gray literature by performing a Google search on 2/4/2021 using the term “non-emergency medical transportation evaluations” and evaluating the first five pages retrieved (58 hits). We also searched the references of included studies and prior systematic reviews (reference mining).

### Study selection

Two team members (PGS and IML) working independently screened the titles of retrieved citations. For titles deemed relevant by at least one person, abstracts were then screened independently in duplicate by team members. All disagreements were reconciled through group discussion. Full-text review was conducted in duplicate by two independent team members (PGS and IML), with any disagreements resolved through discussion. In order to be included, a study had to be an evaluation of an intervention whose focus was on non-emergency transportation access to health resources or non-medical transportation that reported health care outcomes such as missed appointments, costs, and clinical processes and outcomes. As our focus was interventions applicable to the United States, we excluded publications that were describing studies conducted in low- and middle-income countries. Studies whose interventions were multicomponent where the contribution of transportation assistance was small or unstated, such as studies of patient navigators, were excluded [10, 11]. We also excluded studies that were focused on the exercise outcomes of engaging with certain modes of transportation (e.g., cardiovascular health outcomes from increased cycling or walking), and those without health care utilization or outcomes.

### Data extraction and quality assessment

Data extraction was completed in duplicate (by PGS/MMB). All discrepancies were resolved with full group discussion. We abstracted data on the following: study design, sample size, enrolled population, intervention, and outcomes measured. To assess risk of bias we used the Cochrane Risk of Bias Tool [12], the Risk of Bias in Non-randomized Studies of Interventions tool (ROBINS-I) [13], or an adaptation of the National Institutes of Health’s Quality Assessment Tool for Before-After (Pre-Post) Studies with No Control instrument [14].

### Data synthesis and grading

We grouped the interventions into 2 broad categories: those that were just for non-emergency medical transportation (in all cases transportation to clinic/healthcare visits) and interventions that included transportation for non-medical reasons (shopping, etc.) We grouped outcomes into four broad categories: (1) missed clinic visits, (2) healthcare resource utilization, (3) medical costs, and (4) health outcomes.

### Statistical analysis

Odds-ratios (OR) were estimated for each study, comparing pre- to post- intervention for observational studies and intervention to control groups for the randomized control trials. Random effects meta-analysis was performed for outcomes with at least 3 studies. To account for differences in study design types, stratified pooled results are presented along with overall pooled results. Tests of heterogeneity were performed using the I^2^ statistic. Values of the I^2^ statistic closer to 100% represent high degrees of heterogeneity. The Begg rank correlation [15] and Egger regression asymmetry tests were used to examine publication bias. All analyses were conducted in R.4.0.2.

### Rating the certainty of evidence

We based our ratings on the certainty of evidence on the factors considered in the Grading of Recommendations Assessment, Development and Evaluation (GRADE) system [16], and then supplemented by other factors (including mechanistic and parallel evidence) as proposed by Howick and colleagues [17] and as used by the National Academy of Medicine [18].

### Role of the funding source

Funding was provided by Humana. The funder helped set the scope of the review and participated as an author in putting the results in context.

## Results

### Description of the evidence

**Fig. 1:**
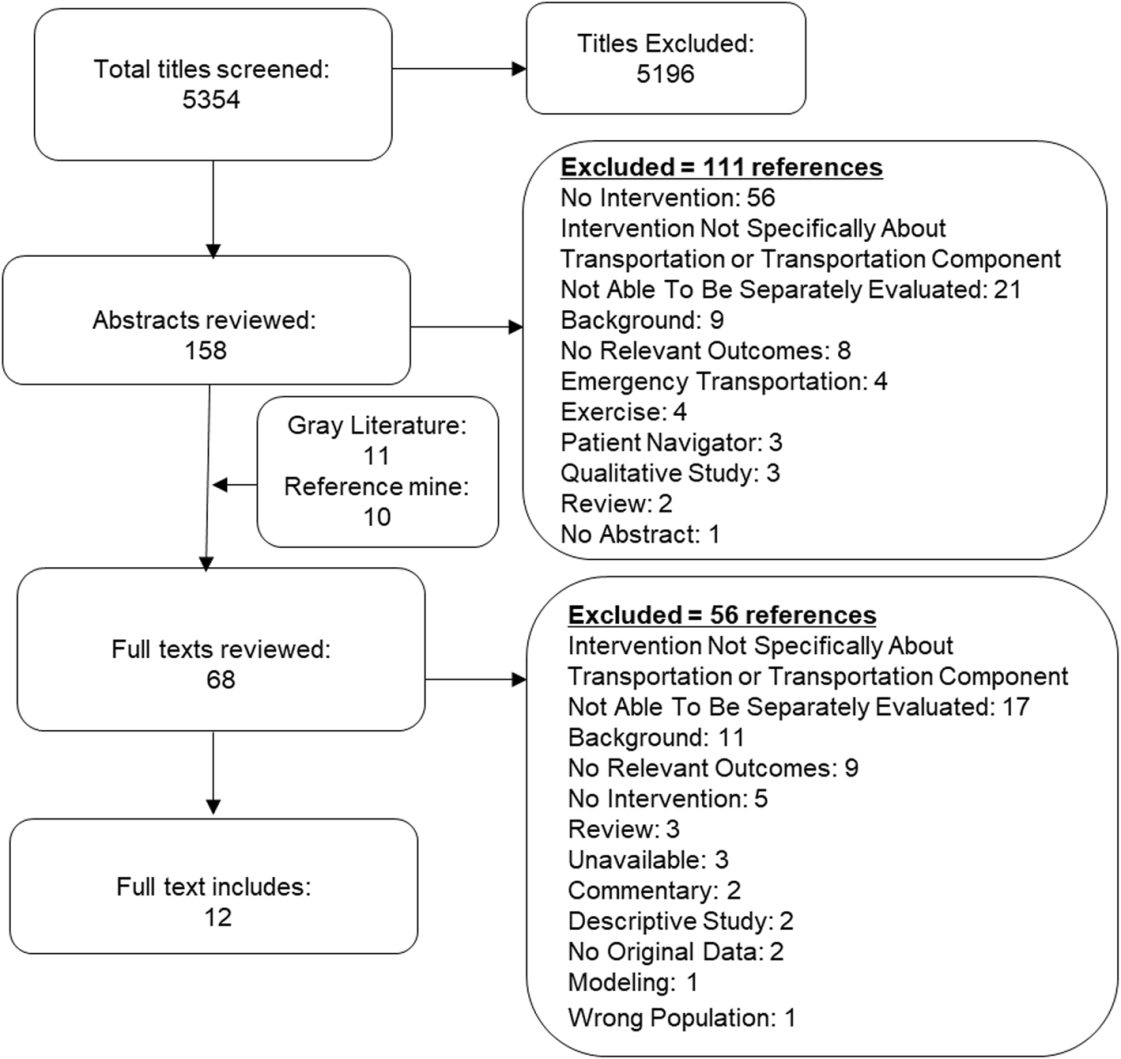
Literature Flow

We identified 5354 potentially relevant citations. Sixty-eight publications underwent full-text review, of which 56 publications were excluded (see Additional file 1: Appendix B). A total of 12 publications were identified as meeting inclusion criteria (see Fig. 1). Of these 12 studies, three were Randomized Controlled Trials (RCTs), one was a controlled clinical trial, and eight were observational studies. One study was conducted in England, the rest were conducted in the United States. Details of included studies are presented in the Evidence Table (see Table 1).

**Table 1:** Evidence Table

Author, Date, ID	Study Design	Sample Size	Enrolled Population	Intervention	Includes non-medical transportation?	Outcomes Measured	Results
Andersen, 2007 [19]	Observational, pre-post	Pre = 61 Post = 61	Urban HIV positive women 82% on Medicaid 91% African American	1–800 phone number to call for free, van ride to doctor’s office	No	Utilization (self-report number of visits, self-report number of missed opportunities)	No change in self-reported overall HIV clinic visits, but decreased self-report of missed appointments from 6 months prior to the intervention(mean 1.92 vs 0.72)
Bryan, 1991 [20]	Observational, pre-post with control	Intervention = 53 Control = 52	Patients who received cancer care and primary care at Wishard Hospital Mean age = 63 7% African American	Both groups received a mailing describing resources to assist with transportation. The intervention group got in addition a telephone consultation to address possible solutions to transportation problems	No	Utilization (missed appointments as measured by electronic health record)	Missed clinic appointments Intervention pre = 23.7%; post = 23.6%; Control pre = 25.5%, post = 39.9%
Chaiyachati, 2018 [21]	Controlled clinical trial	786 (In 2 practices) Intervention = 394 Control = 392	Medicaid adults Mean age = 46 95% African American	Offered rideshare for free transport to clinic	No	Utilization (appointments kept as measured by electronic health record) 7 day or 30-day Emergency Department visits Costs	Missed appointments intervention = 36.5%, control = 36.5%; 7 day ED visits intervention = 2%, control = 1%; mean cost = $14
Chaiyachati, 2018 [22]	Observational Controlled before-and-after	506 (in 2 practices) Intervention = 194 Control = 312	Medicaid adults Mean age = 47 97% African American	Offered rideshare for free transport to clinic	No	Utilization (appointments kept as measured by electronic health record)	Appoints kept intervention pre = 54%, post = 68%; control pre = 60%, post = 51%
Ford, 2019 [23]	RCT	34 Intervention = 29 Control = 5 Participants in 4 primary care practices	Adults greater or equal to 64, not high utilizers and no car access Mean age = 80 100% white	$2000 cash given to clinic to arrange transportation	No	Patient-perceived ease of appointment, cost	Equivalent annual cost per older, socio-disadvantaged older patient without access to a car was lowest in practice with call-stacking system aimed to develop closer links with a community transport provider
Kim, 2009 [24]	Observational Controlled before-and-after	> 50,000	Children with asthma, adults under 65 with DM Medicaid No details of population reported	Transportation brokerage services (profit or non-profit organizations who manage nonemergency medical transportation on a capitated basis)	No	Health, cost, use of transportation services	Statistically significant decreases in monthly NEMT expenditures per person, $18 decrease in mean monthly expenditure per person for both patient groups; decrease in ambulatory care sensitive conditions in diabetics
Marcus, 1992 [25]	RCT	2044	Women with abnormal cervical cytology 79% < age 45 28% African American 41% Hispanic	Bus tickets mailed to women with note that follow-up was needed for cervical cytology result	No	Loss to follow-up	Transportation incentives had significant positive impact on return rates (adjusted odds ratio = 1.48, (95% CI 1.06, 2.06))
Melnikow, 1997 [26]	RCT	Enrolled = 104 (in 5 clinics) Follow-up = 86 Taxi voucher = 34 Blanket coupon = 35 Control = 35	Pregnant women in need of pre-natal care Mean age = 25 53% white	Taxicab voucher for travel to pre-natal appointment	No	Utilization (appointment kept)	Unadjusted odds ratio for keeping first appointment = 0.32 (95% CI 0.12, 0.88)
Saxon, 2019 [27]	Observational, pre-post	Pre = 150 Post = 103	Urban academic health center patients; 60 years of age or older, English-speaking, had a chronic disease, had self-reported transportation barriers Mean age = 72 54% white	3 months of free unlimited ride-share transportation (not just for medical needs)	Yes	Health status (HRQoL; daily step count)	No significant differences between pre-and post-study daily step counts or validated measures of Satisfaction with Life and Geriatric Depression
Vais, 2020a [28]	Observational, pre-post	78 Ride utilizers = 19 Denied transportation issues = 30 Rescheduled < 72 h = 12 Could not be reached by telephone = 17	Gynecology patients who reported difficulties with transportation (excluding obstetrics) Mean age = 36–42 100% African American	Free roundtrip transportation to clinic visit using ride-share	No	Utilization (clinic no-show rate), costs	Weekly no-show rate pre = 27.8%, post = 19.4%; average cost of rides was $32.48
Vais, 2020b [29]	Observational pre-post	86 Ride utilizers = 32 Denied transportation issues = 31 Could not be reached = 23	Patients with sickle cell disease and their caregivers attending a public urban pediatric specialty clinic Mean age = 13 73% African American	Free roundtrip transportation to clinic visit using ride-share	No	Utilization (clinic no-show rate), costs	Decrease in no-show rate from 20.4% to 11.9% using transportation service; total cost of rideshares = $2175; average round trip cost was $67
Whorms, 2021 [30]	Observational, Pre-post	Pre-intervention = 8021 Ride share = 151 Post-intervention non-ride share = 7556	Patients scheduled for MRI at an urban academic health center Mean age 54–60 76% white	Free ride share for patients who spontaneously expressed transportation difficulty in pre-visit reminder telephone call	No	Same day cancellations, timeliness for appointment, cost of rides	No statistically significant difference in same day clinic cancellations (8.1% vs 8.0%); 8 min earlier check-in time for ride share appointment patients, average cost of ride = $17.92

All controlled trials were judged as having at least one domain as being at high risk of bias, but this was because it is not possible to blind participants and personnel to the intervention (see Additional file 1: Appendix Table 1). Two of the four trials were judged as being at low risk of bias for all other domains [25, 26]. Two of the three controlled observational studies were judged as being at low risk of bias in all domains [22, 24]. See Additional file 1: Appendix Table 2. All pre-post studies were limited by one or more of small sample size or loss to follow-up (see Additional file 1: Appendix Table 3).

#### Associations with missed clinic visits and emergency department visits

**Fig. 2:**
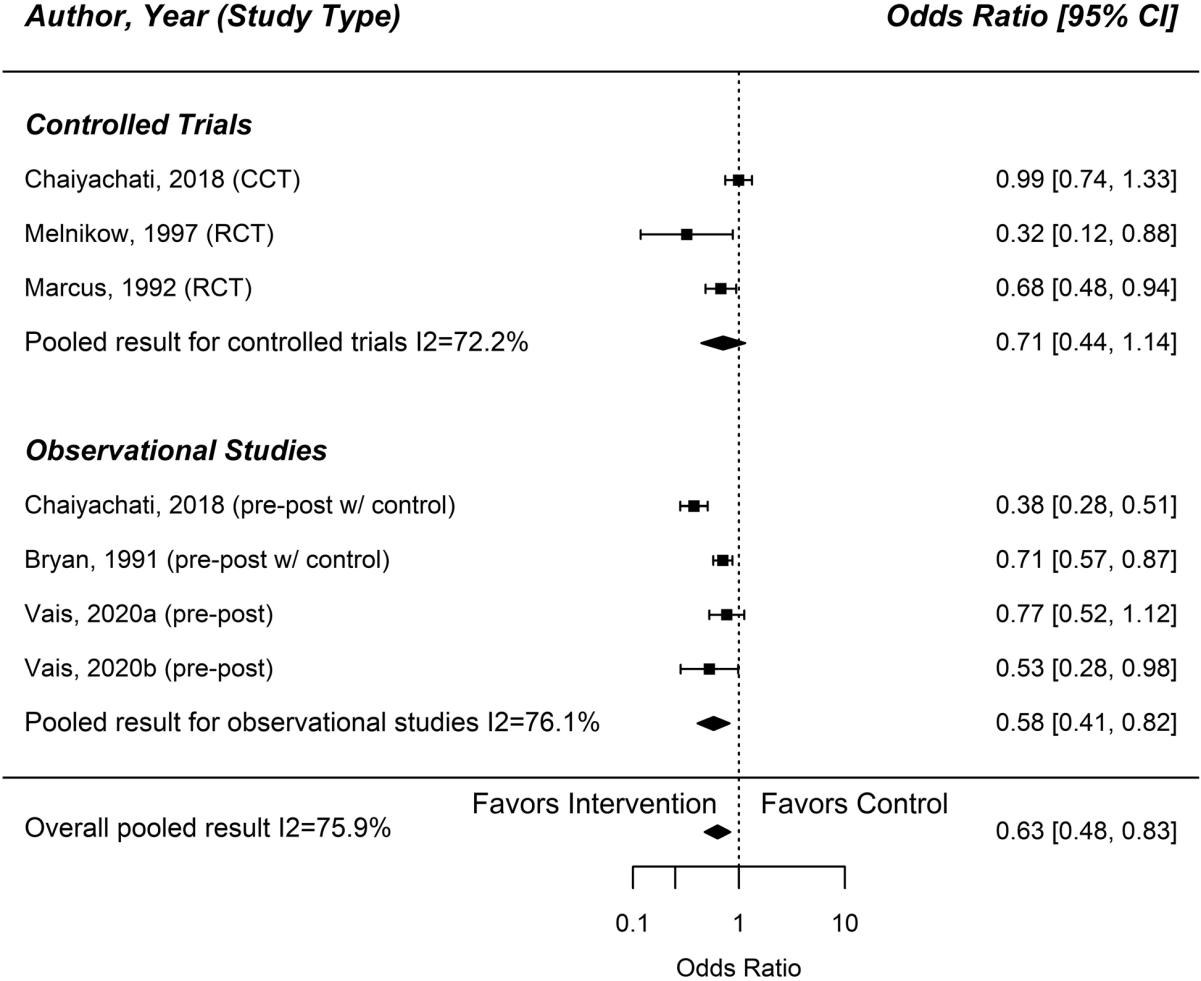
Effect of Interventions to Reduce Transportation Barriers on Missed Appointments

Nine studies reported on made or missed clinic visits [19–22, 25, 27–30]. Three of these studies were controlled trials [21, 25, 26], 1 was a controlled before-and-after study [22], and the rest were pre/post studies [19, 20, 28–30], The enrolled populations were a heterogenous mix of patients with specific conditions (Human Immunodeficiency Virus [HIV], or in need of cervical cytology follow-up, or prenatal care) or patients who were poor, or both. Six studies used as their transportation intervention taxicab or rideshare services (Lyft or Uber) [21, 22, 26, 28–30] 2 used van rides or bus tickets [19, 25], and in one study the intervention was advice and assistance with transportation [20]. One study reported its outcomes as means [19]. Seven studies measured utilization in terms of the proportion of clinic appointments, either missed or kept. The random effects pooled estimate of these seven studies on missed appointments was an odds ratio of 0.63 (95% CI [0.48, 0.83]) in favor of the intervention (see Fig. 2). The I^2^ statistic was 76%. Pooled results from only the three controlled trials (OR = 0.71, 95% CI [0.44, 1.14] were not statistically different from pooled results of the remaining four observational studies (OR = 0.58, 95% CI [0.41, 0.82]. There was no statistical evidence of publication bias (Eggar’s test *p* = 0.24, Begg’s test *p* = 0.38).

The two studies that could not be included in the pooled analysis showed no statistically significant difference in 1) the self-report of the number of HIV visits before and during an intervention that consisted of giving free medical van transportation to and from the clinic for HIV positive women who had been poorly compliant to keeping medical appointments, although the self-reported missed clinic appointments did decline [19]; and 2) the number of patients making same-day cancellations for Magnetic Resonance Imaging (MRI) appointments; in this study only 2% of patients used an offer of a ride-sharing service, making any difference in cancellations due to the intervention very hard to detect.

Two additional studies reported utilization in terms of Emergency Department (ED) visits. The first was a controlled clinical trial that offered 394 Medicaid beneficiaries free rideshare services to come for scheduled clinic visits and compared this to 392 other clinic patients as control [21]. Almost all participants were Black females, the mean age was 46 years. The study found no differences in ED visits at seven or 30 days; however only 20% of eligible patients in the intervention group actually used the rideshare service. The second study assessed the effect of the use of transportation brokerage services for non-emergency medical transportation in adult Medicaid beneficiaries with diabetes, and found that the use of brokerage services did not significantly reduce the probability of ED visits for diabetes [24].

#### Associations with healthcare resource utilization and costs

Six studies reported costs outcomes, one was an RCT [23], one was a controlled before-and-after study [24], three were pre/post studies [24, 29, 30], and one was the controlled clinical trial of rideshare services for Medicaid patients mentioned in the prior Sect. [21] In the latter study, the mean cost of the rideshare per patient who consented was $14.00; in one pre/post study of a ride share for patients scheduled for MRI visits the mean cost was $17.92 [30], and in another pre-post study the mean cost for sickle cell patients was $67 [29], In a study of four general practices in England [23], intervention practices were given an additional 1500 pounds sterling plus technical assistance to improve over the next 6 months their system for making appointments and helping patients with transportation barriers, which included links to community transport, making appointment times convenient for existing bus schedules, providing charging stations for electric scooters, and at one clinic creating an appointment slot for patients requiring taxi services. Two of the three intervention clinics spent £2262 and £930 of additional money on developing their intervention. Staff time devoted to the intervention was estimated at between £112 and £2651.

In a database analysis of the effect of transportation brokerage services on Medicaid expenditures in Georgia and Kentucky, the use of the brokerage service was estimated at decreasing the monthly per person expenditure (inpatient plus outpatient) by about $18 for adults with diabetes and for children with asthma, despite increases in use of health services and prescription drugs (for diabetic patients) [24], All of these interventions were for non-emergency medical transportation to clinic. In one study that included non-medical transportation, discussed below, the cost was $500 per month per patient [27]. One additional evaluation of the use of a ride-sharing program was reported in a blog but could not be included as evidence because it did not report information on the sample sizes, which precluded statistical testing of differences between groups [31].

#### Associations with health outcomes

Two studies assessed health outcomes. One of these studies was the assessment of transportation brokerage services, discussed above [24]. In this study, the use of brokerage services for adult patients with diabetes decreased the probability of having an ambulatory care sensitive condition admission by a statistically significant 0.1 percent a month, whereas for children with asthma there were no such benefits seen. In the only study that assessed an intervention that included non-medical transportation, 150 patients of an urban academic medical center who were over the age of 60 (mean age = 72), had a chronic disease, and reported transportation barriers were offered unlimited ridesharing for 3 months [27]. The mean number of rides during this time period was 69, and the mean cost per subject was $500 per month. Patients also received a device to measure step counts, these did not significantly change from before the intervention to during the intervention. A post-intervention-only assessment of health status, limited by a 31% non-response rate, was reported as showing 92% of subjects having improved quality-of-daily life, but no data are provided in the publication. Also, the published paper reports no significant differences in pre- and post-intervention measures of the Satisfaction with Life survey and the Geriatric Depression Scale; however again no data are reported.

### Certainty of Evidence

We judged the certainty of evidence that providing free Non-Emergency Medical Transportation is associated with a decrease in missed clinic appointments is High, based on the reasonably consistent results seen in controlled trials and observational studies and the strong mechanism that the intervention of providing free transportation might mitigate transportation barriers to care. We judged all other outcomes as being Low or Very Low certainty evidence, due to limitations in study design, and/or execution (see Table 2).

**Table 2:** Certainty of Evidence Table

Intervention and Outcome	Number of studies	Study limitations	Consistency	Precision	Other factors	Overall Certainty of Evidence
***Certainty of Evidence for Missed Clinic Visits***						
Providing free NEMT transportation reduces missed clinic visits	7 (3 controlled trials; 4 observational studies)	Serious	No serious inconsistency	No serious imprecision	Very strong mechanism	High
***Certainty of Evidence for ED Visits***						
Providing free transportation NEMT does not affect ED visit rate	2 (1 controlled trial; 1 observational study)	Serious	No serious inconsistency	Serious imprecision	N/A	Low
***Certainty of Evidence for Costs***						
Providing brokerage service NEMT transport reduces overall healthcare costs	1 (observational study)	Not serious	N/A	No serious imprecision	Data restricted to patients with diabetes and children with asthma	Low
***Certainty of Evidence for Health Outcomes***						
Providing free NEMT improves health outcomes	1 (observational study)	Not Serious	N/A	No serious imprecision	Data restricted to patients with diabetes and children with asthma	Low
Providing free rideshare transport (NMT) improves health outcomes	1 (observational study)	Very Serious	N/A	Serious imprecision	N/A	Very Low

## Discussion

A principal conclusion of this review is that providing transportation means to patients with transportation barriers is associated with a significant reduction in the number of missed clinic appointments. With two exceptions, one a controlled trial where only 20% of eligible patients actually used the ride sharing service [21] and the other a pre-post study where only 2% of patients used the ride sharing service [30], moderately fewer missed clinic appointments was a consistent finding across studies of different design (observational, RCT), different study populations (pregnant women, patient receiving cancer care, poor adults, etc.) and different kinds of transportation options (taxicab vouchers, free bus tickets, free ride-share, etc.).

A second conclusion is that assessments of health outcomes, utilization (other than clinic visits) and costs have been rarely studied. Even in studies that included cost data, this was mostly about the cost of giving the intervention and not about effects on total health care costs. Only one study assessed the effect on health care costs, and this study was not about providing transportation per se, but rather the use of transportation brokers to try and reduce the costs of non-emergency medical transportation. Those results are consistent with a modeling study, not included as empirical evidence, which estimated that with use of “modern” (ride-sharing services) non-emergency medical transportation there may be a savings of $268 per user compared with traditional means of non-emergency medical transportation [32].

A third conclusion is that the effect of providing non-medical transportation – such as to-and from-the grocery store, or shopping for other items needed for daily living, or social engagements – has essentially been unstudied. The one study that evaluated transportation including non-medical needs was limited by methods issues (such as a high loss to follow-up) and a lack of reporting.

These findings have important implications as policymakers, payers, and clinicians search for opportunities to address the health-related social needs of patients and populations. First, reducing missed appointments is assumed to be an important intermediate step towards improved health outcomes. Facilitating access to preventive and primary care services may improve health screening rates, early diagnosis of health conditions, and clinical quality measures. Some data suggest that both the frequency and regularity of primary care provider encounters is associated with better medication adherence and glucose control in patients with diabetes [33]. Thus, under this assumption current evidence justifies expansion of NEMT offerings. NEMT is a required benefit in the Medicaid program to ensure that certain Medicaid beneficiaries have access to transportation to and from medical care. States have flexibility in designing and implementing their NEMT benefits, however there are opportunities to test and standardize the optimal approach. Medicare Advantage plans may offer NEMT to beneficiaries via supplemental benefits. Inherent in these opportunities is the obligation to test, learn and establish the effects on health outcomes. Such work can help establish the optimal model(s) and their effects on health, healthcare resources utilization and overall cost of care.

Increasing access to non-medical transportation may improve health outcomes in a variety of ways, including providing access to grocery stores with more nutritious food and increasing social contacts. Increased access to NMT is listed as an option in a recent Centers for Medicare & Medicaid Services letter to state officials, which says: “States have the option to cover non-medical transportation to enable individuals receiving Medicaid-funded HCBS to gain access to such activities and resources when other options, such as transportation by family, neighbors, friends, or community agencies, are unavailable. Examples include transportation to grocery stores and places of employment.” (https://www.medicaid.gov/federal-policy-guidance/downloads/sho21001.pdf). Medicare Advantage plans may utilize newer pilot opportunities from Center for Medicare & Medicaid Innovation (CMMI), including Special Supplemental Benefits for the Chronically Ill (SSBCI) and Value-Based Insurance Design (VBID). Increased access to NMT should be tested rigorously.

### Limitations

There are several limitations to this review. The main limitation of this review is the quantity and quality of the existing evidence. The majority of studies available were observational, which limits our ability to draw strong conclusions about the effect of the interventions. Nevertheless, the few controlled trials that were identified had results consistent with the observational studies. Secondly, we pooled studies across study designs, but results of pooling studies within study design were consistent and there was no evidence that the controlled trials reported smaller or different results than the observational studies. Thirdly, despite the lack of statistical evidence of publication bias there may be more evaluations of interventions addressing transportation barriers than are reported. The effect of adding in these un-reported studies to those that are published is unknown. Lastly, all of the evidence comes from the USA or England, and the relevance of these results to other contexts can only be inferred.

## Conclusions

In conclusion, the evidence for providing NEMT to patients with transportation barriers shows a clear association with fewer missed clinic visits. Studies of the association of NEMT on health outcomes and costs are thus far too few to draw conclusions. We assume that kept clinic visits should result in better health outcomes, but proving under what circumstances this is correct, and any effect on health care costs, should be a primary focus of future research. NMT is essentially un-studied to date. Studies of the effect on health and financial outcomes of transportation services will help create scalability and sustainability of these services.

## Supplementary Information

**Additional file 1: Appendix Table 1.** Risk of Bias Table. **Appendix Table 2.** ROBINS-I Table. **Appendix Table 3.** Pre-Post Risk of Bias Table. **Appendix A.** Search Strategy. **Appendix B.** Citations for Excluded Studies.

## Data Availability

All data used in these analyses are presented in the Evidence Table or Appendices.

## Abbreviations

CI: Confidence Interval
CMMI: Center for Medicare & Medicaid Innovation
ED: Emergency Department
EMT: Emergency Medical Transport
GRADE: Grading of Recommendations Assessment, Development and Evaluation
HCBS: Home and Community-Based Services
HIV: Human Immunodeficiency Virus
MRI: Magnetic Resonance Imaging
NEMT: Non-Emergent Medical Transport
NMT: Non-Medical Transport
OR: Odds Ration
RCT: Randomized Controlled Trial
ROBINS-I: Risk of Bias in Non-randomized Studies of Interventions
SIREN: Social Interventions Research & Evaluation Network
SSBCI: Special Supplemental Benefits for the Chronically Ill
VBID: Value-Based Insurance Design
