# Supplementary material for: Effect of interventions for non-emergent medical transportation: a systematic review and meta-analysis
Source: BMC Public Health. 2022 Apr 21;22:799. doi: 10.1186/s12889-022-13149-1 (PMC9026972; doi:10.1186/s12889-022-13149-1)
Supplement: Supplementary file 1 — Additional file 1: Appendix Table 1. Risk of Bias Table. Appendix Table 2. ROBINS-I Table. Appendix Table 3. Pre-Post Risk of Bias Table. Appendix A. Search Strategy. Appendix B. Citations for Excluded Studies. [file 12889_2022_13149_MOESM1_ESM.docx]

**Supplementary Tables for Effect of Interventions for Non-Emergent Medical Transportation: A Systematic Review and Meta-Analysis**

Page 2………………………………………………………………………………………………Appendix Table 1. Risk of Bias Table

Page 4………………………………………………………………………………………………..Appendix Table 2. ROBINS-I Table

Page 5…………………………………………………………………………………….Appendix Table 3. Pre-Post Risk of Bias Table

Page 7………………………………………………………………………………………………………..Appendix A. Search Strategy

Page 10…………………………………………………………………………………… Appendix B. Citations for Excluded Studies

**Appendix Table 1. Risk of Bias Table**

| Author, year | Random sequence generation | Allocation concealment | Blinding of participants and personnel | Blinding of outcome assessment | Incomplete outcome data | Selective reporting | Other sources of bias |
| --- | --- | --- | --- | --- | --- | --- | --- |
| Chaiyachati, 2018 (23) | High risk | High risk | High risk | Low risk | Low risk | Low risk | None |
| Ford, 2019 (28) | Low risk | Low risk | High risk | High risk | Low risk | Low risk | High risk; unbalanced intervention and control, too small sample size for randomization to be effective at balancing groups |
| Marcus, 1992 (17) | Low risk | Low risk | High risk | Low risk | Low risk | Low risk | None |
| Melnikow, 1997 (18) | Low risk | Low risk | High risk | Low risk | Low risk | Low risk | None |

**Appendix Table 2. ROBINS-I Table**

| **Author, Year** | **Confounding** | **Selection bias** | **Bias in measurement classification of interventions** | **Bias due to deviations from intended interventions** | **Bias due to missing data** | **Bias in measurement of outcomes** | **Bias in selection of the reported result** |
| --- | --- | --- | --- | --- | --- | --- | --- |
| Bryan, 1991 (22) | High risk | High risk | Low risk | Low risk | Low risk | Low risk | Low risk |
| Chaiyachati, 2018 (19) | Low risk | Low risk | Low risk | Low risk | Low risk | Low risk | Low risk |
| Kim, 2009 (20) | Low risk | Low risk | Low risk | Low risk | Low risk | Low risk | Low risk |

**Appendix Table 3. Pre-Post Risk of Bias Table**

| NIH Quality Assessment Tool for Before-After (Pre-Post) Studies with No Control Group questions | 2. Were eligibility/selection criteria for the study population prespecified and clearly described? | 5. Was the sample size sufficiently large to provide confidence in the findings? | 6. Was the test/service/intervention clearly described and delivered consistently across the study population? | 7. Were the outcome measures prespecified, clearly defined, valid, reliable, and assessed consistently across all study participants? | 9. Was the loss to follow-up after baseline 20% or less? Were those lost to follow-up accounted for in the analysis? |
| --- | --- | --- | --- | --- | --- |
| Anderson, 2007 (21) | Yes | No | Yes | Yes | Low |
| Saxon, 2019 (24) | Yes | No | Yes | Yes | High |
| Vais, 2020a (22) | Yes | No | Yes | Yes | Low |
| Vais, 2020b (27) | Yes | No | Yes | Yes | Low |
| Whorms 2021 (26) | Yes | No | Yes | Yes | Low |

**Appendix A. Search Strategy**

**DATABASE SEARCHED & TIME PERIOD COVERED:**

**PubMed – From inception to 01/14/2022**

**SEARCH STRATEGY: 3716 results**

(((((((((((Barrier*[tiab] OR access*[tiab] OR facilitat*[tiab] OR utilization[tiab]) AND (((care[tiab] AND health[tiab]) OR healthcare[tiab]) OR "Appointments and Schedules"[Mesh] OR appointment*[tiab] OR (resources[tiab] AND community[tiab]) OR nutrition[tiab] OR (food[tiab] AND (health[tiab] OR healthy[tiab])) OR groceries[tiab] OR "Exercise"[Mesh] OR exercise[tiab] OR "physical activity"[tiab] OR "physically active"[tiab])) OR "Health Services Accessibility"[Mesh] OR "Healthcare Disparities"[Mesh] OR "Health Equity"[Mesh]) AND ("Transportation"[Mesh:noexp] OR "Transportation of Patients"[Mesh:noexp] OR Transportation[tiab] OR transit[tiab]))) NOT (animal[mesh] NOT human[mesh]))) NOT (child[mesh] NOT adult[mesh]))) NOT ((("Africa"[mesh] OR "Latin America"[mesh] OR "South America"[mesh] OR "Central America"[mesh] OR "Asia"[Mesh] OR "Mexico"[Mesh] OR "Islands"[Mesh] OR "Oceania"[Mesh]) NOT ("Canada"[Mesh] OR "United States"[Mesh] OR "Europe"[mesh]))))) AND English[Language]

**PubMed – From inception to 01/14/2022**

**SEARCH STRATEGY: 77 results**

((Rideshare[tiab] OR ridesharing[tiab] OR uber[tiab] OR lyft[tiab]) AND (transport OR transportation))

**-------------------------------------------------------------------------------------------------------------------------------**

**DATABASE SEARCHED & TIME PERIOD COVERED:**

**Cochrane Reviews/Trials – From inception to 01/14/2022**

**SEARCH STRATEGY: 163 results**

**(((((((Barrier*:ti,ab,kw OR access*:ti,ab,kw OR facilitat*:ti,ab,kw OR utilization:ti,ab,kw) AND** (((care:ti,ab,kw AND health:ti,ab,kw) OR healthcare:ti,ab,kw) OR [mh "Appointments and Schedules"] OR appointment*:ti,ab,kw OR (resources:ti,ab,kw AND community:ti,ab,kw) OR nutrition:ti,ab,kw OR (food:ti,ab,kw AND (health:ti,ab,kw OR healthy:ti,ab,kw)) OR groceries:ti,ab,kw OR [mh "Exercise"] OR exercise:ti,ab,kw OR "physical activity":ti,ab,kw OR "physically active":ti,ab,kw)) OR [mh "Health Services Accessibility"] OR [mh "Healthcare Disparities"] OR [mh "Health Equity"]) AND ([mh ^"Transportation"] OR [mh ^"Transportation of Patients"] OR Transportation:ti,ab,kw OR transit:ti,ab,kw)))NOT ([mh animal] NOT [mh human])) NOT ([mh child] NOT [mh adult])) NOT (([mh "Africa"] OR [mh "Latin America"] OR [mh "South America"] OR [mh "Central America"] OR [mh "Asia"] OR [mh "Mexico"] OR [mh "Islands"] OR [mh "Oceania"]) NOT ( [mh "Canada"] OR [mh "United States"] OR [mh "Europe"])

OR

((Rideshare OR ridesharing OR uber OR lyft):ti,ab,kw AND (transport OR transportation))

**-------------------------------------------------------------------------------------------------------------------------------**

**DATABASE SEARCHED & TIME PERIOD COVERED:**

**Web of Science** (SCI-EXPANDED, SSCI, A&HCI) **– From inception to 1/14/2022**

**SEARCH STRATEGY: 1304**

TS=(Barrier* OR access* OR facilitat* OR utilization) AND (TS=(care NEAR/2 health) OR TS=(healthcare OR appointment*) OR TS=(resources NEAR/2 community) OR TS=(nutrition) OR (TS=(food) AND TS=(health NEAR/2 healthy))) OR (TS=(groceries OR exercise OR "physical activity" OR "physically active")) OR (TS=("health services" NEAR/2 accessibil*)) OR (TS=(healthcare NEAR/2 disparit*)) OR (TS=(health NEAR/2 equit*)) AND TS=(Transportation OR transit)

**-------------------------------------------------------------------------------------------------------------------------------**

**DATABASE SEARCHED & TIME PERIOD COVERED:**

**University of California, San Francisco Social Interventions Research & Evaluation Network database** on 01/26/2021 using their Social Determinant of Health ‘Transportation’ filter categorization.

**SEARCH STRATEGY: 94 results**

**Appendix B. Citations for Excluded Studies**

Intervention Not Specifically About Transportation or Transportation Component Not Able To Be Separately Evaluated, n=17

1. Berkowitz, S.A., et al., Mechanisms Associated with Clinical Improvement in Interventions That Address Health-Related Social Needs: A Mixed-Methods Analysis. Popul Health Manag, 2019. 22(5): p. 399-405.

2. Esperat, M.C., et al., Transformacion Para Salud: a patient navigation model for chronic disease self-management. Online J Issues Nurs, 2012. 17(2): p. 2.

3. Frank, S., et al., Transportation innovation to aid Parkinson disease trial recruitment. Contemp Clin Trials Commun, 2019. 16: p. 100449.

4. Gottlieb, L.M., H. Wing, and N.E. Adler, A Systematic Review of Interventions on Patients' Social and Economic Needs. Am J Prev Med, 2017. 53(5): p. 719-729.

5. Kaplan, C.P., et al., Improving follow-up after an abnormal pap smear: results from a quasi-experimental intervention study. J Womens Health Gend Based Med, 2000. 9(7): p. 779-90.

6. Krieger, J., et al., Linking community-based blood pressure measurement to clinical care: a randomized controlled trial of outreach and tracking by community health workers. Am J Public Health, 1999. 89(6): p. 856-61.

7. Niccols, A. and W. Sword, “New Choices” for substance using mothers and their children: Preliminary evaluation. Journal of Substance Use, 2005. 10(4): p. 239-251.

8. Percac-Lima, S., et al., A culturally tailored navigator program for colorectal cancer screening in a community health center: a randomized, controlled trial. J Gen Intern Med, 2009. 24(2): p. 211-7.

9. Pruitt, Z., et al., Expenditure Reductions Associated with a Social Service Referral Program. Popul Health Manag, 2018. 21(6): p. 469-476.

10. Raven, M.C., et al., An intervention to improve care and reduce costs for high-risk patients with frequent hospital admissions: a pilot study. BMC Health Serv Res, 2011. 11: p. 270.

11. Shier, G., et al., Strong social support services, such as transportation and help for caregivers, can lead to lower health care use and costs. Health Aff (Millwood), 2013. 32(3): p. 544-51.

12. Taylor, V., et al., A clinic‐based mammography intervention targeting inner‐city women. Journal of general internal medicine, 1999. 14(2): p. 104-111.

13. Taylor, V.M., et al., A randomized controlled trial of interventions to promote cervical cancer screening among Chinese women in North America. J Natl Cancer Inst, 2002. 94(9): p. 670-7.

14. Tierney, W.M., et al., Restricting medicaid payments for transportation: effects on inner-city patients' health care. Am J Med Sci, 2000. 319(5): p. 326-33.

15. Tsega, S. and H.J. Cho, The Reality of Accessing Transportation for Health Care in New York City. JAMA Netw Open, 2019. 2(6): p. e196856.

16. Wilber, K.H., et al., Partnering managed care and community-based services for frail elders: the care advocate program. J Am Geriatr Soc, 2003. 51(6): p. 807-12.

17. Yue, D., et al., Enabling Services Improve Access To Care, Preventive Services, And Satisfaction Among Health Center Patients. Health Aff (Millwood), 2019. 38(9): p. 1468-1474.

Background, n=11

1. NEMP for Medicaid Patients, in Medicaid Non-Emergency Medical Transportation. West Virginians for Affordable Health Care.

2. Adelberg, M., et al., Non-Emergency Medical Transportation: Findings from a Return on Investment Study. 2018.

3. Adelberg, M. and M. Simon, Non-Emergency Medical Transportation: Will Reshaping Medicaid Sacrifice An Important Benefit? 2017, Health Affairs.

4. Crable, E.L., et al., Interventions to increase appointment attendance in safety net health centers: A systematic review and meta-analysis. J Eval Clin Pract, 2020.

5. Fraade-Blanar, L. and C. Whaley, Non-Emergency Medical Transportation in the Time of COVID-19. 2020, The RAND Blog.

6. Hennein, L. and A.G. de Alba Campomanes, Association of a Health Coaching and Transportation Assistance Intervention at a Free Ophthalmology Homeless Shelter Clinic With Follow-up Rates. JAMA Ophthalmol, 2021. 139(3): p. 311-316.

7. Powers, B., S. Rinefort, and S. Jain, Shifting Non-Emergency Medical Transportation To Lyft Improves Patient Experience And Lowers Costs. 2018, Health Affairs Blog.

8. Samuel, P.S., et al., Benefits and Quality of Life Outcomes From Transportation Voucher Use by Adults With Disabilities. Journal of Policy and Practice in Intellectual Disabilities, 2013. 10(4): p. 277-288.

9. Solomon, E.M., et al., Impact of Transportation Interventions on Health Care Outcomes: A Systematic Review. Med Care, 2020. 58(4): p. 384-391.

10. Wolfe, M.K. and N.C. McDonald, Innovative health care mobility services in the US. BMC Public Health, 2020. 20(1): p. 906.

11. Wolfe, M.K., N.C. McDonald, and G.M. Holmes, Transportation Barriers to Health Care in the United States: Findings From the National Health Interview Survey, 1997-2017. Am J Public Health, 2020. 110(6): p. 815-822.

No Relevant Outcomes, n=9

1. Bellamy, G.R., et al., Getting from here to there: evaluating West Virginia's rural nonemergency medical transportation program. J Rural Health, 2003. 19 Suppl: p. 397-406.

2. Bove, A.M., S.T. Gough, and L.R.M. Hausmann, Providing no-cost transport to patients in an underserved area: Impact on access to physical therapy. Physiother Theory Pract, 2019. 35(7): p. 645-650.

3. Eisenberg, Y., et al., Rideshare Transportation to Health Care: Evidence from a Medicaid Implementation. The American Journal of Managed Care, 2020. 26(9).

4. Knight, K.E., Federally Qualified Health Centers Minimize the Impact of Loss of Frequency and Independence of Movement in Older Adult Patients through Access to Transportation Services. J Aging Res, 2011. 2011: p. 898672.

5. MacLeod, K.E., et al., The implementation cost of a safety-net hospital program addressing social needs in Atlanta. Health Serv Res, 2021. 56(3): p. 474-485.

6. Martin, S.L., J. Wood, and S. Soule, A Volunteer Program in Maine to Transport Community Members to Health Care Appointments. Prev Chronic Dis, 2020. 17: p. E77.

7. Mielenz, T.J., et al., Fall Risk Reduction Program Paired with a Transportation Program in an Underserved, Urban Minority Community: A Qualitative Evaluation. J Aging Res, 2019. 2019: p. 2719290.

8. Safaei, J., A ride to care--a non-emergency medical transportation service in rural British Columbia. Rural Remote Health, 2011. 11(2): p. 1637.

9. Wortman, Z., E.C. Tilson, and M.K. Cohen, Buying Health For North Carolinians: Addressing Nonmedical Drivers Of Health At Scale. Health Affairs, 2020. 39(4): p. 649-654.

No Intervention, n=5

1. Alley, D.E., et al., Accountable Health Communities--Addressing Social Needs through Medicare and Medicaid. N Engl J Med, 2016. 374(1): p. 8-11.

2. Li, F. and C. Kajetan Wyczalkowsk, Can subsidized transportation options slow diabetes progression? 2019, Systems for Action.

3. Powers, B.W., S. Rinefort, and S.H. Jain, Nonemergency Medical Transportation: Delivering Care in the Era of Lyft and Uber. Jama, 2016. 316(9): p. 921-2.

4. Toscos, T., et al., Identifying Successful Practices to Overcome Access to Care Challenges in Community Health Centers: A "Positive Deviance" Approach. Health Serv Res Manag Epidemiol, 2018. 5: p. 2333392817743406.

5. van Schalkwyk, M.C.I. and J.S. Mindell, Current issues in the impacts of transport on health. Br Med Bull, 2018. 125(1): p. 67-77.

Review, n=3

1. Bambra, C., et al., Tackling the wider social determinants of health and health inequalities: evidence from systematic reviews. J Epidemiol Community Health, 2010. 64(4): p. 284-91.

2. O'Brien, N., et al., Health System Features That Enhance Access to Comprehensive Primary Care for Women Living with HIV in High-Income Settings: A Systematic Mixed Studies Review. AIDS Patient Care STDS, 2018. 32(4): p. 129-148.

3. Starbird, L.E., et al., A Systematic Review of Interventions to Minimize Transportation Barriers Among People with Chronic Diseases. J Community Health, 2019. 44(2): p. 400-411.

Unavailable, n=3

1. Borders, S., N. Chaudhuri, and J. Dyer, An assessment of non-emergency medical transportation (NEMT) barriers in Texas and their impacts on access to and utilization of EPSDT services, in 136st APHA Annual Meeting and Exposition 2008. 2008.

2. Southworth, F., D.P. Vogt, and T.R. Curlee, Rural transit systems benefits in Tennessee: methodology and an empirical study. Environment and Planning a-Economy and Space, 2005. 37(5): p. 861-875.

3. Thomas, L.V. and K.R. Wedel, Nonemergency medical transportation and health care visits among chronically ill urban and rural medicaid beneficiaries. Soc Work Public Health, 2014. 29(6): p. 629-39.

Commentary, n=2

1. Callahan, M., N. Cooper, J. Sisto Gall, and J. Yoo. 2021. 'Industry-informed perspectives on the benefits of rideshare-based medical transportation', Am J Manag Care, 27: 271-72.

2. Eisenberg, Y., R. Owen, and C. Crabb. 2021. 'Reply to "industry-informed perspectives on the benefits of rideshare-based medical transportation"', Am J Manag Care, 27: 272-73.

Descriptive Study, n=2

1. Holt, Wexler & Farnam, LLP. 2007. "Assessment of Non-Emergency Medical Transportation in Upper Litchfield County." In.: Connecticut State Office of Rural Health.

2. Myers, A. 2015. "Non-Emergency Medical Transportation: A Vital Lifeline for a Healthy Community." In.: National Conference of State Legislatures.

No Original Data, n=2

1. Parfaite-Claude, D. Lyft Releases Data Showing its Non-Emergency Medical Transport Services Help Medicaid Beneficiaries. 2020; Available from: <https://www.ancor.org/newsroom/news/lyft-releases-data-showing-its-non-emergency-medical-transport-services-help-medicaid>. Accessed 19 February 2021.

2. Slomski, A., Ridesharing Services May Reduce Alcohol-Associated Car Crashes. Jama, 2021. 326(5): p. 378.

Modeling, n=1

1. Rochlin, D. H., et al. (2019). "Economic Benefit of "Modern" Nonemergency Medical Transportation That Utilizes Digital Transportation Networks." Am J Public Health 109(3): 472-474.

Wrong Population, n=1

1. Borders, S., et al., Devolution's policy impact on non-emergency medical transportation in State Children's Health Insurance Programs. Soc Work Public Health, 2011. 26(2): p. 137-57.
